# Homebound older adults who live independently in rural Japan: Prevalence and contributing factors during the COVID-19 pandemic^☆^

**DOI:** 10.1016/j.pmedr.2024.102640

**Published:** 2024-02-05

**Authors:** Saori Koizumi, Akiko Ohta, Midori Kamei

**Affiliations:** Division of Public Health, Department of Social Medicine, Faculty of Medicine, Saitama Medical University, 38 Morohongo Moroyama-machi, Iruma-gun, Saitama 350-0495, Japan

**Keywords:** COVID-19, Homebound, Independently, Japan, Older adults, Prevalence, Rural health

## Abstract

Being homebound is a phenomenon of confining older adults to their homes owing to health, social, and psychological factors. During the COVID-19 pandemic, people were requested to refrain from going out to prevent infection. Consequently, the homebound status of older adults was influenced by social and environmental factors, resulting in an increase in the number of homebound older adults during the pandemic. This study aimed to determine the homebound prevalence and related factors among homebound older adults during the COVID-19 pandemic. In 2021, a cross-sectional study conducted in rural Saitama, Japan, included 1,020 participants aged 65 years and above who did not have long-term care insurance certification and were independent in instrumental activities of daily living. Herein, homebound individuals were defined as those who went out once a week or less. The relationships of homebound status with demographic, health, social, and psychological factors in older adults were examined. The prevalence of homebound independent older adults was estimated as 10.4 % (6.6 % males, 13.8 % females). Homebound status was significantly associated with one or more medical histories (odds ratio [OR] = 1.98, 95 % confidence interval [CI] = 1.00–3.90), no social or family roles (OR = 1.95, 95 % CI = 1.09–3.48), and no hobbies (OR = 1.84, 95 % CI = 1.02–3.34). Establishing social or family roles and promoting participation in hobbies may prevent older adults from being homebound. The social environment, which changed during the pandemic, should be improved to encourage older adults to go out.

## 1 Introduction

Homebound status is described as limiting the living space of older adults to their homes (Schirghuber and Schrems, 2021, Ko and Noh, 2021). In Japan, the number of homebound older adults was first reported in the early 1980s (Yasumura, 2003). Most Japanese researchers have defined being homebound using the frequency of going out for a certain period (e.g., less than or equal to once a week) (Ko and Noh, 2021). Earlier studies have shown a higher risk of mortality (Cohen-Mansfield et al., 2010, Jacobs et al., 2018, Soones et al., 2017) and disabilities (Fujita et al., 2006, Kawamura et al., 2005) among homebound older adults. Thus, the Japanese Ministry of Health, Labor and Welfare established care prevention programs in 2006 to prevent frailty and disability in older adults and introduced the Kihon Checklist (Arai and Satake, 2015) to screen homebound adults and prevent them from being disabled.

Based on various definitions of the term “homebound” used in earlier studies, the prevalence of homebound status ranges from 3.5 % to 39.8 % (Lee et al., 2022). In Japan, according to the narrow definition of homebound status, i.e., individuals who go out of their home once a week or less, the prevalence of homebound status ranges from 7.5 % to 11.7 % (Hanno City, 2021, Tsuji et al., 2021, Kawamura et al., 2005). Based on sex, the homebound prevalence was higher in females than in males (Ganguli et al., 1996, Jing et al., 2017, Negrón-Blanco et al., 2016, Ornstein et al., 2015). In terms of age, the prevalence of homebound older adults increased with aging (Ganguli et al., 1996, Jing et al., 2017, Ornstein et al., 2015). Based on regional differences in Japan, the lower the population density, the higher the homebound prevalence (Hirai et al., 2008).

Before the COVID-19 pandemic, the concept of being homebound was an enduring condition in which the living space was confined to the home owing to health, social, and psychological factors (Jing et al., 2017, Negrón-Blanco et al., 2016, Schirghuber and Schrems, 2021, Shinkai et al., 2005a). Among healthy older adults, being homebound was especially related to social factors, such as having no close friends and no social activities (Shinkai et al., 2005b, Watanabe et al., 2007). However, during the pandemic, being homebound was considered a temporary condition in addition to the conventional concept, as older adults could not go out of their homes owing to enforced restrictions, such as lockdowns. Several global surveys have reported a decrease in opportunities for face-to-face interactions and an increase in the prevalence of homebound individuals (Ankuda et al., 2021, Cabinet Office, 2020). In Japan, an increase in the prevalence of homebound individuals was documented during the pandemic (Tanaka et al., 2023) despite the declaration of a state of emergency by the Japanese Government, which encouraged people to stay at home without imposing penalties (Cabinet Secretariat, 2020). However, there is a paucity of research on homebound older adults in Japan during the COVID-19 pandemic, highlighting the need for further investigations in this area. Since May 2023, the Japanese government has downgraded COVID-19 to a “common infectious disease” (Ministry of Health, Labour and Welfare of Japan, 2023). The concept of being homebound during the post-COVID-19 pandemic period is the same as that during the pre-pandemic period because there are no mandatory lockdowns. However, the reasons for being homebound include conventional factors and changing social conditions or psychological aspects owing to the pandemic. To understand the impact of the pandemic on older adults and to help encourage them to go out will be needed.

This study aimed to determine the homebound prevalence and associated factors among independent older adults in rural Japan during the COVID-19 pandemic.

## 2 Materials and methods

### 2.1 Study design and participants

A cross-sectional study was conducted in 2021, wherein the data were collected from all residents aged 30 years and above, except for institutionalized residents, in three districts of Hanno City, Saitama, Japan. Hanno City, located in southwest Saitama Prefecture, boasts abundant natural surroundings, with approximately 75 % of the area covered by forests. This research focused on three rural districts within this city, which are predominantly inhabited by long-time residents. Notably, the population of individuals aged 65 years and above accounted for 40.1 %, 40.5 %, and 46.7 % of the total population in these three districts, respectively, surpassing the national average of 28.9 % in 2021 (Statistics Bureau of Japan, 2021).

**Fig. 1:**
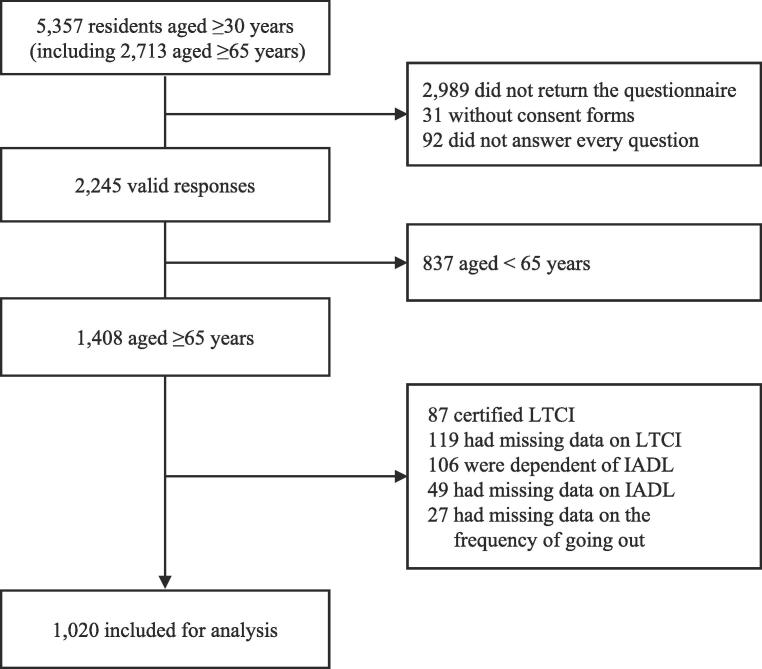
Flowchart illustrating the participant selection process, including excluded cases. Participants were chosen based on age, LTCI certification, IADL performance, and the data on the frequency of going out. LTCI: Long-term care insurance. IADL: Instrumental activities of daily living.

In October 2021, self-administered questionnaires were mailed to 5,357 residents (including 2,713 residents aged ≥65 years), and 2,368 residents responded to the questionnaires (response rate, 44.2 %). In total, 31 individuals without consent forms and 92 individuals who did not answer every question were excluded, resulting in 2,245 valid responses (valid response rate, 41.9 %). Of the 1,408 valid responses from individuals aged ≥65 years (valid response rate, 51.9 %), 1,020 independent older adults answered the question regarding the frequency of going out and were thus eligible for the current study (Fig. 1).

Independent older adults were defined as those aged 65 years and above who did not have long-term care insurance [LTCI] certification and were independent in instrumental activities of daily living [IADL]. LTCI certification is an insurance system for citizens wherein they can receive care and support from the society as a whole. Municipalities provide LTCI certification based on the screening results of physical and cognitive functions (Ministry of Health, Labour and Welfare of Japan, 2002). Herein, IADL was assessed using a subscale of the Tokyo Metropolitan Institute of Gerontology Index of Competence (Koyano et al., 1991), which has adequate validity and reliability for measuring the high-level competence of community-dwelling older adults (Fujihara et al., 2019, Koyano et al., 1991, Yamashita et al., 2020). IADL assessment comprises five questions related to using public transport, shopping, preparing meals, paying bills, and banking. Full scores for IADL indicate independent living within the community. Some earlier studies in Japan have defined independent older adults as those without LTCI certification (Kabayama et al., 2018, Miyashita et al., 2021). However, owing to concerns regarding the impact of the pandemic on older adults who can live and go outside without assistance, the definition of independent older adults in this study was revised to encompass those who had no LTCI certification and were independent in IADL.

### 2.2 Measures

#### 2.2.1 Dependent variable

The dependent variable was homebound status. All independent older adults were categorized as homebound or non-homebound. Based on the methodology employed by Shinkai et al. (2005b), the following question was used to determine the homebound status: “How often do you go out for work, fieldwork, strolling, shopping, hospital visitation, and social activities?” The responses included ≥5 times a week, 2–4 times a week, once a week, and rarely. Participants who answered “once a week” or “rarely” were categorized as homebound, whereas those who answered “≥5 times a week” or “2–4 times a week” were categorized as non-homebound.

#### 2.2.2 Independent variables

The independent variables included demographic characteristics and health, psychological, and social factors. Earlier studies have shown that the antecedents of being homebound were health, psychological, and social factors, supporting this study’s framework (Hiyama et al., 2022, Jing et al., 2017, Negrón-Blanco et al., 2016, Shinkai et al., 2005a, Shinkai et al., 2005b, Wakayama et al., 2016).

Demographic characteristics included sex, age, household structure, and work status. Sex categories included male and female. Based on age, the participants were classified as 65–74 years old and 75 years and above. The household structures of participants were categorized into different types as living alone, married couple, two-generation household, three-generation household, brothers and sisters, and others. The household structures were categorized as living alone or with others, in accordance with an earlier study (Tsuji et al., 2021). The work status was either worker or non-worker.

In this study, health factors encompassed both medical history and cognitive function. Medical history was classified into two groups: individuals with one or more medical histories and those with no medical history. Cognitive function was assessed using the Kihon Checklist-Cognitive Function [KCL-CF] (Arai and Satake, 2015, Tomata et al., 2017). KCL-CF assessment consists of three questions, each offering two choices. KCL-CF scores, ranging from 0 to 3, were assigned based on the respondents’ answers to these three questions. A score of one or higher indicated a decline in cognitive function. Participants were categorized as having either “maintained” or “decreased” cognitive function.

Social factors included participating in neighborhood associations and hobbies as well as establishing social or family roles. In Japan, neighborhood associations are voluntary groups engaged in various community activities focusing on their respective neighborhoods (Pekkanen, 2005). Regarding participation in neighborhood associations (categorized as often, sometimes, rarely, never), “often” or “sometimes” response was categorized as participation, and “rarely” or “never” response was categorized as nonparticipation. Social or family roles were classified based on whether individuals played such roles, which involved caring for family members, friends, or neighbors. Hobbies were classified into two categories: individuals with one or more hobbies and those with no hobbies. Participants were asked to select multiple answers from a list of 39 different hobbies (Supplementary data 2).

Psychological factors included subjective economic status and self-rated health. These categorized variables were combined into binary based on earlier studies. For subjective economic status (very good, good, fair, poor, very poor), “very poor” or “poor” response was defined as “poor,” and the rest were defined as “good” (Ejiri et al., 2018). For self-rated health (very good, good, fair to poor, poor), “very good” or “good” was categorized as “good” and “fair to poor” or “poor” was categorized as “poor” (Shinkai et al., 2005b).

### 2.3 Statistical analyses

Participants were categorized as homebound or non-homebound. The prevalence of “homebound” individuals was calculated by dividing the number of homebound cases by the total number of individuals in the study population. The demographic characteristics and health, social, and psychological factors of homebound and non-homebound individuals were compared using chi-square tests. The association of homebound status with health, social, and psychological factors was determined using multivariable logistic regression models. Model 1 presented the data after adjusting for sex, age, household structure, and work status. Model 2 involved the forced entry of variables such as sex, age, medical history, cognitive function, neighborhood associations, social or family roles, hobbies, subjective economic status, and self-rated health. No multicollinearity was detected among these variables (VIF < 2.5) (Johnston et al., 2018). A statistical significance level of 0.05 was considered for the analyses. All statistical analyses were conducted using IBM SPSS Statistics 26.0 for Windows (IBM Corp, Armonk, NY, USA).

### 2.4 Ethical considerations

This study was approved by the Ethical Committee of Saitama Medical University (Approval Number: Dai2021–014; July 27, 2021). Consent forms were sent to all participants along with questionnaires explaining the study purposes and ethical considerations, including voluntary participation, data management methods, and appropriate use of study results. Participants were only included in the study if they returned the consent form with the questionnaire.

## 3 Results

### 3.1 Homebound prevalence

As shown in Table 1, among 1,020 independent older adults, 106 were homebound (32 males, 74 females), with a prevalence of 10.4 % (6.6 % males, 13.8 % females). When participants were classified according to age, 8.5 % of individuals aged 65–74 years and 13.1 % of those aged 75 years and above were found to be homebound.

**Table 1:** Prevalence of homebound individuals in rural Japan in 2021.

			Sex						Age					
Total	Homebound		Total	Homebound		Total	Homebound		Total	Homebound		Total	Homebound	
N	n	(%)	N_m_	n_m_	(%)	N_f_	n_f_	(%)	N_65–74_	n_65–74_	(%)	N_75+_	n_75+_	(%)
1,020	106	(10.4)	485	32	(6.6)	535	74	(13.8)	600	51	(8.5)	420	55	(13.1)

N_m_: Total number of maes.

n_m_: Number of homebound males.

N_f_: Total number of females.

n_f_: Number of homebound females.

N_65–74_: Total number of participants aged 65–74 years.

n_65–74_: Number of homebound individuals aged 65–74 years.

N_75+_: Total number individuals aged 75 years and above.

n_75+_: Total number of homebound individuals aged 75 years and above.

### 3.2 Demographic characteristics and health, social, and psychological factors

Table 2 shows the demographic characteristics as well as health, social, and psychological factors of participants based on their homebound status. Overall, 69.8 % of homebound individuals were females, and 51.9 % were 75 years and above. Regarding household structure, 21.6 % of homebound individuals and 15.0 % of non-homebound individuals lived alone. With regard to the work status, 6.9 % of homebound individuals and 30.0 % of non-homebound individuals were workers. For health factors, 84.8 % of homebound individuals demonstrated one or more medical histories and 35.7 % of homebound individuals exhibited decreased cognitive function. In terms of social factors, of the homebound individuals, 75.5 % participated in neighborhood associations, 69.6 % played social or family roles, and 76.4 % enjoyed hobbies. Regarding psychological factors, 67.6 % of homebound individuals reported good subjective economic status and 73.3 % of homebound individuals have good self-rated health.

**Table 2:** Demographic characteristics and health, social, and psychological factors by homebound status.

		Total		Homebound		Non-homebound			
		(N=1,020)		(n=106)		(n=914)			
		N	(%)	n	(%)	n	(%)	*P*-value	
**Demographic characteristics**									
Sex									
	Male	485	(47.5)	32	(30.2)	453	(49.6)	<0.001	***
	Female	535	(52.5)	74	(69.8)	461	(50.4)		
Age									
	65–74	600	(58.8)	51	(48.1)	549	(60.1)	0.022	*
	75^+^	420	(41.2)	55	(51.9)	365	(39.9)		
Household structure									
	Alone	157	(15.7)	22	(21.6)	135	(15.0)	0.113	
	With others	843	(84.3)	80	(78.4)	763	(85.0)		
Work status									
	Worker	277	(27.6)	7	(6.9)	270	(30.0)	<0.001	***
	Non-Worker	725	(72.4)	94	(93.1)	631	(70.0)		

**Health factors**									
Medical history									
	None	246	(24.6)	16	(15.2)	230	(25.6)	0.022	*
	One or more	756	(75.4)	89	(84.8)	667	(74.4)		
Cognitive function									
	Maintained	666	(67.5)	63	(64.3)	603	(67.9)	0.496	
	Decreased	320	(32.5)	35	(35.7)	285	(32.1)		

**Social factors**									
Participation in neighborhood associations									
	Participation	816	(82.3)	77	(75.5)	739	(83.0)	0.074	
	Nonparticipation	176	(17.7)	25	(24.5)	151	(17.0)		
Social or family roles									
	Playing a role	794	(80.5)	71	(69.6)	723	(81.8)	0.004	**
	No roles	192	(19.5)	31	(30.4)	161	(18.2)		
Hobbies									
	One or more	874	(85.7)	81	(76.4)	793	(86.8)	0.006	**
	None	146	(14.3)	25	(23.6)	121	(13.2)		

**Psychological factors**									
Subjective economic status									
	Good	759	(75.2)	71	(67.6)	688	(76.1)	0.072	
	Poor	250	(24.8)	34	(32.4)	216	(23.9)		
Self-rated health									
	Good	876	(86.2)	77	(73.3)	799	(87.7)	<0.001	***
	Poor	140	(13.8)	28	(26.7)	112	(12.3)		

Chi-square tests.

*P < 0.05, **P < 0.01, ***P < 0.001.

Bivariate analysis results (Table 2) demonstrated significant differences in all demographic characteristics except for household structure showed significant differences when participants were grouped by homebound status. Regarding health, social, and psychological factors, being homebound was associated with having one or more medical histories, no social or family roles, no hobbies, and poor self-rated health.

### 3.3 Association of homebound status with health, social, and psychological factors

Table 3 shows the association of homebound status with health, social, and psychological factors using logistic regression analysis. In Model 1, all data were adjusted for sex, age, household structure, and work status. Homebound status was significantly associated with one or more medical histories (Odds ratio [OR] = 2.17, 95 % confidence interval [CI] = 1.15–4.11), no social or family roles (OR = 1.91, 95 % CI = 1.12–3.25), no hobbies (OR = 1.96, 95 % CI = 1.14–3.36), and poor self-rated health (OR = 2.59, 95 % CI = 1.57–4.27). Model 2 involved the forced entry of variables such as sex, age, medical history, cognitive function, neighborhood associations, social or family roles, hobbies, subjective economic status, and self-rated health. Homebound status was significantly associated with one or more medical histories (OR = 1.98, 95 % CI = 1.00–3.90), no social or family roles (OR = 1.95, 95 % CI = 1.09–3.48), and no hobbies (OR = 1.84, 95 % CI = 1.02–3.34).

**Table 3:** Association among health, social, and psychological factors and homebound status.

	Category	Total		Homebound							
		(N=1,020)		(n=106)		Model 1^a^			Model 2^b^		
		N	(%)	n	(%)	OR	95%CI		OR	95%CI	
**Health factors**											
Medical history	One or more	756	(75.4)	89	(84.8)	2.17	1.15–4.11	*	1.98	1.00–3.90	*
Cognitive function	Decreased	320	(32.5)	35	(35.7)	1.16	0.73–1.84		0.89	0.54–1.47	

**Social factors**											
Participation in neighborhood associations	Nonparticipation	176	(17.7)	25	(24.5)	1.46	0.88–2.45		1.36	0.78–2.39	
Social or family roles	No roles	192	(19.5)	31	(30.4)	1.91	1.12–3.25	*	1.95	1.09–3.48	*
Hobbies	None	146	(14.3)	25	(23.6)	1.96	1.14–3.36	*	1.84	1.02–3.34	*

**Psychological factors**											
Subjective economic status	Poor	250	(24.8)	34	(32.4)	1.59	0.99–2.54		1.51	0.90–2.54	
Self-rated health	Poor	140	(13.8)	28	(26.7)	2.59	1.57–4.27	***	1.54	0.86–2.75	

OR: odds ratio, 95 %CI: 95 % confidence interval.

*P < 0.05, ***P < 0.001.

a Adjusted for sex, age, household structure and work status.

b In addition to Model 1, medical history, cognitive function, participation in neighborhood associations, social or family roles, hobbies, subjective economic status, and self-rated health were included.

## 4 Discussion

### 4.1 Homebound prevalence

Herein, the homebound prevalence among participants aged 65 years and above was 10.4 %. The homebound prevalence in a 2019 survey conducted in the same city and using the same homebound definition as those before the pandemic was 11.7 % (Hanno City, 2021). Homebound prevalence did not increase during the COVID-19 pandemic. However, other studies have reported contradictory results. A 2020 survey in Japan revealed that 41 (13.9 %) of 281 participants became newly homebound as a result of the first stay-at-home order (Tanaka et al., 2023). In the US, the homebound prevalence of individuals aged 70 years and above was more than doubled, from approximately 5.0 % during 2011–2019 to 13.0 % in 2020 (Ankuda et al., 2021). Considering that this study was conducted in 2021, which was the second year of the COVID-19 pandemic, it is reasonable to assume that the homebound prevalence did not increase substantially during this period. A Japanese study involving older adults indicated that the physical activity of participants declined during the first year of the pandemic but exhibited a recovery trend in the second year (Yamada and Arai, 2022). Another study revealed that mobility patterns initially decreased significantly in the early stages of the pandemic in Japan, but a relatively high level of activeness was observed as people started adapting to new circumstances (Tsuboi et al., 2022). Regarding the social situation in Japan during the pandemic, the government did not issue any stay-at-home order during this study period. Furthermore, approximately 80 % of Japanese older adults had received their second dose of the COVID-19 vaccination (Prime Minister’s Office of Japan, 2023). These conditions might have encouraged older individuals to go outside the home.

To determine the impact of enforced restrictions on older adults during the pandemic, this study included participants who were independent in IADL. This study participants were much healthier than those in the 2019 survey. No earlier studies have focused on homebound prevalence among IADL-independent older adults. Regarding healthy adults, the homebound prevalence was 7.5 %–10.0 % among ADL-independent older adults (Kawamura et al., 2005, Tsuji et al., 2021, Watanabe et al., 2011), 4.8 %–6.8 % among older adults who could go out alone (Shinkai et al., 2005a, Shinkai et al., 2005b), and 13.8 % among ambulatory older adults aged 75 years and above (Yokoyama et al., 2005). Thus, the homebound prevalence in this study cannot be compared with that in earlier studies owing to differences in the study populations. However, the prevalence of 10.4 % in this study (13.1 % among adults aged 75 years and above) is not much higher than that reported before the pandemic.

The questionnaire in this study asked for the frequency of going out for specific purposes (e.g., work, fieldwork, strolls, shopping, hospital visitation, and social activities), which were not used in the 2019 survey. Mentioning the specific purposes when inquired about the frequency of going out may lower the prevalence of homebound status (Hirai and Kondo, 2022). Thus, this homebound prevalence may be understated.

### 4.2 Factors associated with homebound status

This study showed that neither social or family roles nor hobbies were related to homebound status. Earlier studies have indicated that social factors, such as less interaction with close friends, no family roles, no practice of walking or exercising, and no hobbies, were related to being homebound among healthy older adults (Shinkai et al., 2005b, Watanabe et al., 2007). These findings were consistent with those of earlier studies.

From a list of individuals including spouses, children, siblings, parents, grandchildren, relatives, neighbors, friends, and others, this study participants had the option to select multiple individuals for whom they care. This study revealed that most participants who played social or family roles primarily cared for their spouses or children (Supplementary data 1). Considering the restrictions on meeting nonfamily members during the COVID-19 pandemic, engaging in family roles may have contributed to promoting outings. In rural areas of Japan, residents used to traditionally meet and assist one another before the pandemic. However, only a small number of the study participants reported caring for friends or neighbors. As playing social roles for older adults is known to prevent homebound status (Shinkai et al., 2005b, Watanabe et al., 2007), neighborly relationships similar to those before the pandemic are expected to continue post pandemic.

On the contrary, participation in neighborhood associations, which is a group activity, was not associated with homebound status. Shinkai et al. revealed that one of the reasons for older adults to become homebound was nonparticipation in group activities (Shinkai et al., 2005b). As mass gatherings were not allowed during the pandemic, the responses revealed that participation in neighborhood associations did not promote outings.

In this study, participants were given the opportunity to select multiple hobbies from a list of 39 hobbies. Notably, several participants preferred hobbies such as fieldwork and walking, which are activities that can be enjoyed individually (Supplementary data 2). Participants who were engaged in hobbies by themselves during the pandemic may have been more inclined to go out.

In Japan, municipalities and prefectures used to organize hobby and volunteer activities aimed at promoting active and healthy lifestyles among older adults even before the COVID-19 pandemic (Ministry of Health, Labour and Welfare of Japan, 2017). These initiatives provide opportunities for older adults to engage in various hobbies and contribute to their overall well-being. The results of this study suggest that providing the same support as that before the pandemic can be useful for independent older adults, which includes establishing social and family roles and promoting participation in hobbies. Unfortunately, some activities that were discontinued during the COVID-19 pandemic have not yet been resumed. Thus, the resumption of such activities and provision of opportunities for older adults to socialize are expected in the future.

### 4.3 Limitations and strengths

This study has several limitations. First, as this is a cross-sectional study, a causal relationship could not be established. Second, the questionnaire did not contain factors that were previously linked to being homebound, including hearing impairments (Jing et al., 2017), depression (Cohen-Mansfield et al., 2010, Jing et al., 2017, Shinkai et al., 2005a), and self-efficacy for going out (Yamazaki et al., 2021). Third, this research was conducted in rural areas; therefore, the results may reflect only regional characteristics. Fourth, the lack of information regarding ethnicity suggests that most participants were Japanese, limiting the generalizability of this findings to foreign populations.

Despite these limitations, this study has certain strengths. First, the valuable insights into homebound prevalence among 1,020 older adults during the COVID-19 pandemic were provided. There are limited studies highlighting homebound prevalence in Japan during the pandemic, and the number of participants in this study, although not extensive, contributes to the existing body of knowledge (Tanaka et al., 2023). Second, this study elucidated the factors associated with homebound older adults who maintained their IADL during the pandemic.

## 5 Conclusions

This study revealed that 10.4 % of independent older adults in this study area were homebound during the COVID-19 pandemic. Establishing social or family roles and promoting participation in hobby activities can prevent older adults from being homebound. The similar social factor approach as that before the pandemic can be useful for independent older adults. The social environment, which has changed during the pandemic, should be improved to encourage older adults to go out.

## Funding

This research did not receive any specific grant from funding agencies in the public, commercial, or not-for-profit sectors.

## CRediT authorship contribution statement

**Saori Koizumi:** Writing – original draft, Visualization, Methodology, Investigation, Formal analysis, Data curation, Conceptualization. **Akiko Ohta:** Writing – review & editing, Methodology, Investigation, Conceptualization. **Midori Kamei:** Writing – review & editing, Supervision, Project administration, Investigation.

## Declaration of competing interest

The authors declare that they have no known competing financial interests or personal relationships that could have appeared to influence the work reported in this paper.

## Data Availability

The data that has been used is confidential.
